# Toll-like receptor 3 pathway restricts Marek’s disease virus infection

**DOI:** 10.18632/oncotarget.20003

**Published:** 2017-08-07

**Authors:** Haitao Zou, Ruixue Su, Jing Ruan, Hongxia Shao, Kun Qian, Jianqiang Ye, Aijian Qin

**Affiliations:** ^1^ Ministry of Education Key Lab for Avian Preventive Medicine, Yangzhou University, Yangzhou, Jiangsu, 225009, P.R. China; ^2^ Jiangsu Co-Innovation Center for Prevention and Control of Important Animal Infectious Diseases and Zoonoses, Yangzhou, Jiangsu, 225009, P.R. China; ^3^ Jiangsu Key Lab of Zoonosis, Yangzhou, Jiangsu, 225009, P.R. China

**Keywords:** Marek’s disease virus, toll-like receptor 3, poly (I:C), interferon-β, inflammatory cytokines

## Abstract

Marek’s disease virus (MDV) is an α-herpesvirus that causes immune suppression and T lymphoma in chickens. Toll-like receptor 3 (TLR3) is critical for the host immune response against MDV infection. Previously, our team demonstrated that pre-treatment of TLR3 agonist poly (I:C) inhibited Marek’s disease virus infection in chicken embryo fibroblasts (CEFs). However, whether TLR3 inhibits the aggravation of MDV infection is unknown. In the current study, we found that TLR3 activation in MDV-infected CEFs effectively inhibited virus spread. Using pharmacological approaches, we revealed that pro-inflammatory cytokines and interferon-β induced by TLR3 could restrict Marek’s disease virus infection. This study contributes to elucidating the function and mechanism of the TLR3 pathway in host immune responses against MDV infection.

## INTRODUCTION

The innate immune system recognizes pathogen-associated molecular patterns (PAMPs) through pattern recognition receptors (PRRs). Toll-like receptors (TLRs) are a typical class of PRRs in the innate immune response against viral infections. Toll-like receptor 3 (TLR3), a member of the TLR family, is a type I intracellular transmembrane protein that contains a large leucine-rich repeat (LRR) in its extracellular region and a Toll-IL-1 receptor (TIR) homology signalling domain in its cytoplasmic region [1]. Double-stranded RNA (dsRNA) binding leads to TLR3 dimerization and activation of its TIR domain, which recruits the adapter molecule TIR-containing adaptor molecule-1 (TICAM-1; also called TRIF) [2]. TRIF recruits either TNF receptor-associated factor 6 (TRAF6) and receptor-interacting protein 1 (RIP1) to activate NF-κB, or TRAF3 to induce interferon regulatory factor 3 (IRF3), mediating innate immune responses to limit viral replication [3].

An increasing body of evidence has indicated that TLR3 responds to dsRNA derived from viral particles or infected cells and subsequently initiates signal transduction to induce a cytokine response, such as the production of interleukelin-6 (IL-6) and interferon-β (IFN-β) [4, 5]. Activation of TLR3 impairs the replication of several RNA viruses, including dengue virus (DENV), human immunodeficiency virus (HIV), influenza virus and respiratory syncytial virus (RSV), in different cell types through induction of cytokines and interferon [6–9]. In studies of herpesvirus, stimulation with TLR3 agonist was also found to inhibit herpes simplex virus (HSV) replication in human neuronal cells and genital epithelial cells [10, 11]. Furthermore, pretreatment with a TLR3 agonist induced innate immunity against HSV infection in a mouse model [12, 13]. Marek’s disease virus (MDV) is an avian α-herpesvirus that causes immune suppression and T lymphoma in chickens [14]. MDV is a strict cell-associated virus and, thus, has a long and complex viral replication cycle [15]. MDV infection was reported to be inhibited in chicken embryo fibroblasts (CEFs) pretreated with poly (I:C), a TLR3 agonist [16]. However, it is unknown whether activation of TLR3 can restrict the aggravation of MDV infection.

In the present study, we first showed that activation of TLR3 inhibited the aggravation of MDV infection. We then demonstrated that both IFN-β and pro-inflammatory cytokines induced by TLR3 activation contributed to the restriction of MDV infection in CEFs.

## RESULTS

### Activation of TLR3 restricted the development of MDV infection

**Figure 1:**
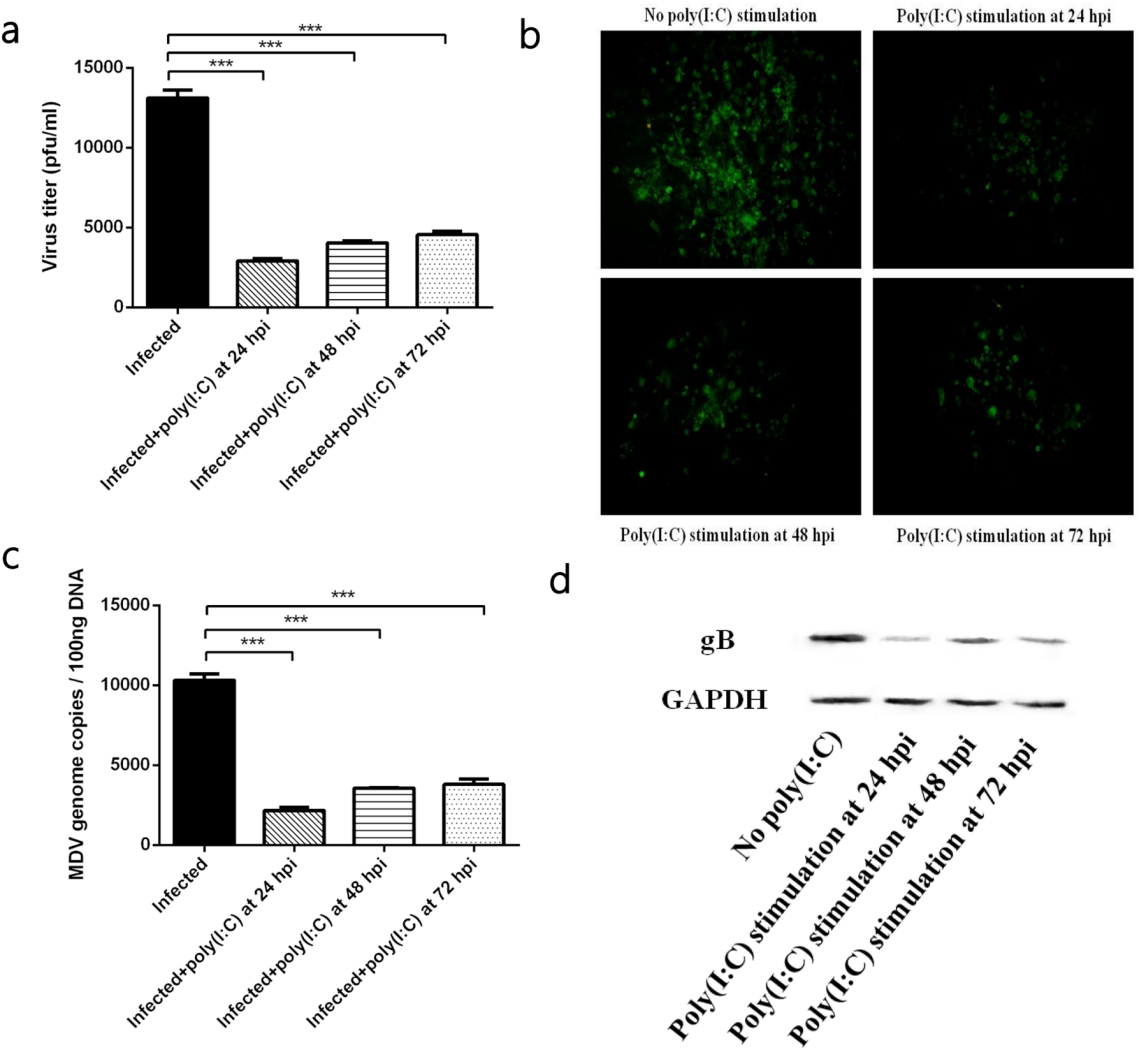
The effect of TLR3 activation on MDV infection **(a)** Viral titre of the RB1B strain in CEF cells either untreated or stimulated with poly (I:C) at 96 hpi. **(b)** Plaques of the RB1B strain in CEF cells either untreated or stimulated with poly (I:C) at 96 hpi. **(c)** Viral genome copies of the RB1B strain in CEF cells either untreated or stimulated with poly (I:C) at 96 hpi. **(d)** Level of the viral protein gB in the RB1B strain in CEF cells either untreated or stimulated with poly (I:C) at 96 hpi. An asterisk (*), double asterisk (**) or triple asterisk (***) indicates p <0.05, 0.01<p <0.05 or p <0.01 for statistical difference from the controls, respectively. Error bars represent standard errors.

In MDV-infected CEFs, we found that TLR3 activation effectively restricted the development of MDV infection. The TLR3 agonist poly (I:C) was added to infected cells at 24, 48 and 72 hpi, respectively. At 96 hpi, viral titre and viral genomic copies in infected CEFs stimulated with poly (I:C) for 24, 48 and 72h were significantly lower than those in the un-stimulated group (Figure 1a, 1c). At the same time, the plaques size in infected CEFs stimulated with poly (I:C) for 24, 48 and 72h were also smaller than those in the control group (Figure 1b). Furthermore, western blot assays showed that the expression of the viral protein gB in infected CEFs stimulated with poly (I:C) for 24, 48 and 72h was less than that in the untreated group (Figure 1d). Similar inhibitory effect of poly (I:C) were found in CEFs infected with MDV virulent strain GA (data not shown). These results suggested that TLR3 activation restricted MDV infection regardless of when that activation occurred.

### Poly (I:C) stimulation increased transcripts associated with the TLR3 pathway in MDV-infected CEFs

**Figure 2:**
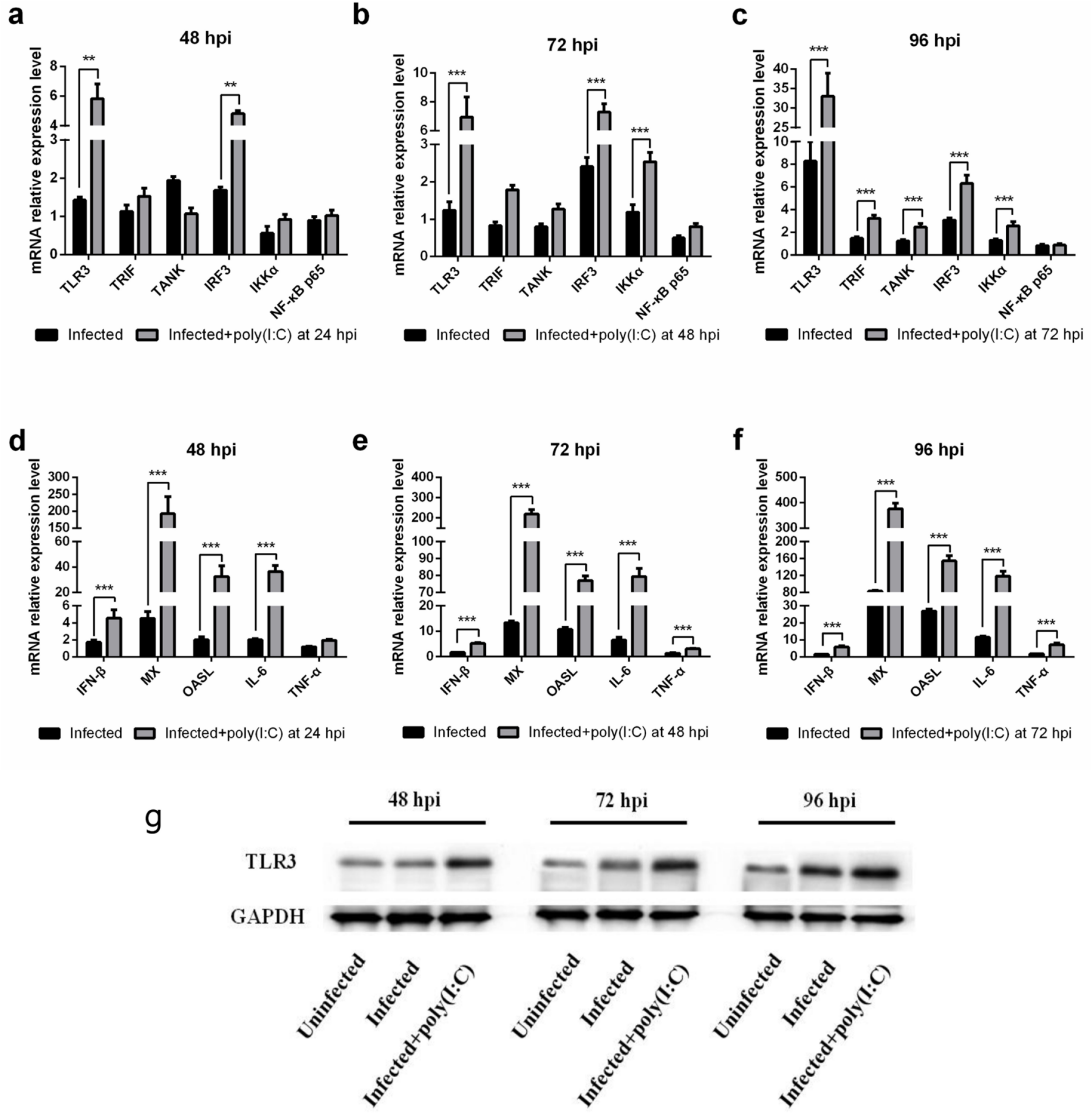
The effect of TLR3 activation on expression of genes associated with the TLR3 pathway in MDV-infected CEF Cells Transcriptional level of genes associated with the TLR3 pathway in MDV-infected CEFs either untreated or stimulated with poly (I:C) at 48 hpi **(a, d)**, 72 hpi **(b, e)** and 96 hpi **(c, f)**. **(g)** Level of TLR3 protein in MDV-infected CEFs either untreated or stimulated with poly (I:C) at 48 hpi, 72 hpi and 96 hpi. An asterisk (*), double asterisk (**) or triple asterisk (***) indicates p <0.05, 0.01<p <0.05 or p <0.01 for statistical difference from the controls, respectively. Error bars represent standard errors.

We investigated the expression pattern of several genes associated with the TLR3 pathway by real-time PCR. *TLR3*, *IRF3* and *IKKα* were substantially increased in the stimulated groups compared with those in the control group (Figure 2a-2c). TLR3 increase in the stimulated groups was further confirmed by western blot analysis (Figure 2g). *IRF3* and *IKKα* were related to IFN-β and inflammatory cytokine production, respectively. Similarly, the mRNA levels of *IFN-β*, *IL-6* and *TNF-α* were significantly increased in MDV-infected CEFs stimulated with poly (I:C) (Figure 2d-2f). These data suggested that TLR3 activation induced IFN-β and inflammatory cytokines to restrict MDV infection.

### Both IFN-β and inflammatory cytokines induced by TLR3 contributed to MDV infection inhibition

**Figure 3:**
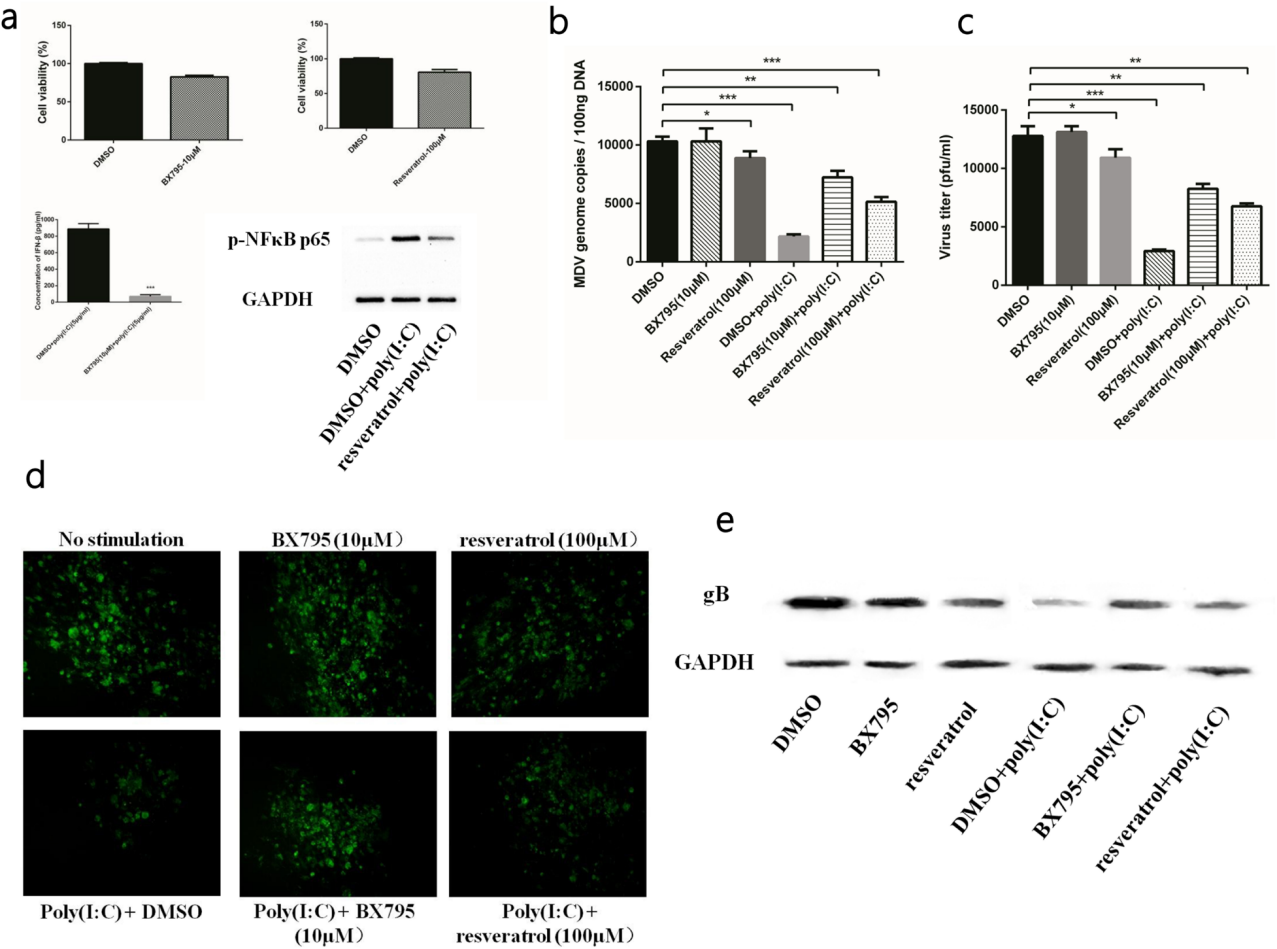
TLR3 activation induced IFN-β and inflammatory cytokines to restrict MDV infection **(a)** The inhibitory effect and cell viability of two inhibitors in CEFs. **(b)** Viral titre of the RB1B strain in poly (I:C)-stimulated CEF cells either untreated or treated with inhibitors at 96 hpi. **(c)** Viral genome copies of the RB1B strain in poly (I:C)-stimulated CEF cells either untreated or treated with inhibitors at 96 hpi. **(d)** Plaques of the RB1B strain in poly (I:C)-stimulated CEF cells either untreated or treated with inhibitors at 96 hpi. **(e)** Level of the viral protein gB in the RB1B strain in poly (I:C)-stimulated CEF cells either untreated or treated with inhibitors at 96 hpi. An asterisk (*), double asterisk (**) or triple asterisk (***) indicates p <0.05, 0.01<p <0.05 or p <0.01 for statistical difference from the controls, respectively. Error bars represent standard errors.

To confirm that the anti-MDV effect was mediated by IFN-β and inflammatory cytokines, we used two specific small-molecule inhibitors, BX795 and resveratrol, to interfere with the induction of IFN-β and inflammatory cytokines, respectively. The BX795 at a concentration of 10μM of and resveratrol at a concentration of 100μM showed no significant negative effect on CEFs in CCK8 assay (Figure 3a). And the results of ELISA and western blot confirmed that BX795 and resveratrol were able to effectively inhibit the production of IFN-β and inflammatory cytokines, respectively (Figure 3a). Viral titre and number of viral genome copies showed that both BX795 and resveratrol attenuated rather than abolished the antiviral effect of poly (I:C) (Figure 3b, 3c). At the same time, the plaques in the inhibiter-treated groups were larger than those in the untreated group, but smaller than those in the group infected with MDV only (Figure 3d). Similar results were also found in the viral gene expression analysis (Figure 3e). Overall, the data suggested that IFN-β and inflammatory cytokines induced by the TLR3 pathway contributed to inhibiting MDV infection.

## DISCUSSION

TLR3 has been identified as an important innate immune-recognition receptor for dsRNA. Many studies have shown the potential antiviral function of TLR3 in herpesvirus infection [10-12, 17]. Previously, we demonstrated that MDV infection was inhibited in CEFs pretreated with poly (I:C) [16]. Moreover, our current study showed that activation of TLR3 also restricted the development of MDV infection. Thus, TLR3 activation is a key mediator of the host immune response against MDV regardless of when that activation occurred.

Binding of TLR3 and dsRNA promotes signal transduction through the adapter protein TRIF to activate the transcription factors IRF3 and NF-κB, which promote the expression of IFN-β and inflammatory cytokines. Several studies have shown that inflammatory cytokine and IFN-β induction by TLRs can help the host impair DENV-2, HIV, and RSV infections [6–8]. BX795 is a relatively specific inhibitor of TBK1 and IKKε which block the phosphorylation, nuclear translocation, and transcriptional activity of IRF3, to inhibit the production of IFN-β [18]. Resveratrol specifically inhibits TRIF signaling in the TLR3 pathway by targeting TBK1 and RIP1 in TRIF complex, resulting in inhibition of pro-inflammatory cytokines expression [19]. In chemical inhibitors assays, our results indicated that suppressing either inflammatory cytokines or IFN-β significantly attenuated the anti-MDV effect of poly (I:C). These data suggested that the antiviral effects of TLR3 in MDV infection are through the production of IFN-β and inflammatory cytokines. Previous studies showed that type I IFN protected cell and host from MDV infection, which was consistent with our finding [20, 21]. Several IFN-stimulated genes (ISG) induced by type I IFN have been identified and characterized in chicken [22]. The IFN positive-feedback loop is important for inducing and sustaining antiviral innate immune responses. In this study, we observed that synthetic dsRNA stimulation elevated two typical ISGs, Mx and OASL, in MDV-infected CEFs. These results indicated that TLR3 was involved in the IFN-β response.

Moreover, no significant change in *IFN-β* expression was detected during MDV infection, while *IFN-β* was increased in MDV-infected CEFs after poly (I:C) stimulation. These data suggested that MDV inhibits IFN-β production. Several studies have identified a variety of mechanisms for blocking IFN-β production during α-herpesvirus infection. The infected cell protein 0 encoded by HSV-1 inhibits IRF3- and IRF7-mediated activation of the IFN-β gene [23]. The HSV-1 ubiquitin-specific protease UL36 and serine/threonine kinase US3 inhibit IFN-β production by deubiquitinating TRAF3 and hyperphosphorylating IRF3, respectively [24, 25]. The MDV genes located in the UL and US segments are largely homologous to, and arranged collinearly with, those of HSV-1 [15]. Thus, there are likely to be similar mechanisms for blocking IFN-β production during MDV infection, which can further explain the immune suppression caused by this infection.

Inflammatory cytokines are indispensable in host defence against α-herpesvirus. IL-18 is associated with the rapid activation of NK cells to restrict HSV-1 replication in the lung [26]. Mast cells inhibit HSV-1 infection through TNF-α and IL-6 production [27], and IL-6 deficiency enhances susceptibility to HSV-1 in mice [28]. Similarly, IL-1β, IL-6 and IL-18 were associated with an immune response to MDV, but this has yet not been directly confirmed. The current results indicated that inflammatory cytokines induced by TLR3 inhibit MDV infection. Moreover, we revealed that inflammatory cytokines were directly related to the anti-MDV effects. Unfortunately, it is unclear which type of inflammatory cytokine most efficiently restricts MDV infection. Several chicken inflammatory cytokines have been expressed with biological activity *in vitro* [29, 30]. Thus, it is possible to test the effect of different inflammatory cytokines on MDV infection, which may promote the development of novel MDV vaccines or anti-MDV drugs.

Collectively, our results have helped elucidate the function and mechanism of the TLR3 pathway in the host immune response against MDV.

## MATERIALS AND METHODS

### Cells and virus

Primary CEFs were prepared from 10-day-old specific-pathogen-free (SPF) embryos obtained from Merial Vital (Laboratory Animal Technology Co., Ltd, Beijing, China) and cultured in DMEM supplemented with 5% FBS at 37°C in 5% CO_2_. The very virulent RB1B strain of MDV was maintained in our laboratory.

### Reagents and antibodies

The TLR3 agonist poly (I:C) (HMW) and the inhibitors (BX795 and resveratrol) were purchased from InvivoGen (San Diego, CA, USA). Mouse monoclonal anti-GAPDH antibody was from Abcam (Cambridge, MA, USA). Rabbit polyclonal anti-TLR3 antibody was from Novus Biologicals (Littleton, Colorado, USA). The mouse anti-MDV monoclonal antibody BD8 and rabbit polyclonal anti-NFκB antibody were maintained by our laboratory. The ELISA kit for chicken IFN-β was from Nanjing Senbeijia Biological Technology Co., Ltd (Nanjing, China).

### Virus infection and cell treatment

Secondary CEFs were seeded in 6-well plates and cultured for 18-24 h following infection with the RB1B strain at an MOI=0.001. The TLR3 agonist poly (I:C) was added at a final concentration of 5 μg/ml to infected cells at 24, 48 and 72 h post infection (hpi). The infected cells were collected at 48, 72 and 96 hpi [16].

Secondary CEFs infected with the RB1B strain at an MOI=0.01 were cultured for 24 h. MDV-infected CEFs were pretreated with inhibitors (either 10 μM BX795 or 100 μM resveratrol) for 2 h following stimulation with poly (I:C). The infected cells were collected at 96 hpi.

### Real-time PCR

The total RNA and MDV genomic DNA from infected cells were isolated using TRIzol Reagent (Invitrogen) following the manufacturer’s instructions. The cDNA synthesis was performed using a PrimeScript™ RT reagent Kit (TaKaRa, Dalian, China). Real-time PCR was performed using SYBR Premix ExTaq II (TaKaRa Dalian, China) in an ABI 7500 real-time PCR system (Applied Biosystems, CA, USA). The sequences of specific primers used for real-time PCR were previously published elsewhere [31]. Gene expression levels were normalised to the expression of chicken 18S snRNA. The changes in gene expression were calculated using the 2^−ΔΔCt^ method.

### Virus titration

The infected cells were centrifuged and re-suspended in 1ml medium. Infected-cell suspension was made for serial 10-fold dilution. Secondary CEFs were seeded in 96-well plates and cultured over night, and then these cells were infected with MDV in different dilution. Each dilution group has twelve replicates. After incubation at 37°C in 5% CO_2_ for 96h, the cells were treated as described in plaque assay. The numbers of plaques in different dilution were counted.

### Virus Plaque compared by Immunofluorescence

The cells were fixed with an acetone: ethanol solution (3:2) for 10 min and washed once with PBS. After the fixed cells were blocked with 1% BSA in PBS, they were incubated with the mouse anti-MDV monoclonal antibody BD8 for 45 min at 37°C. After three washes with PBS, the cells were incubated with FITC-conjugated goat anti-mouse IgG (Sigma-Aldrich, USA) for another 45 min. After three washes with PBS, the cells were examined under a fluorescence microscope. The plaque sizes between different groups were compared.

### Western blot analysis

Cell pellets were washed once with PBS and lysed with RIPA buffer containing protease inhibitors on ice. The samples were loaded with 5× denaturing sample buffer and separated by 12% SDS-PAGE. The proteins were transferred to a 0.2-μm nitrocellulose (NC) membrane. The mouse monoclonal anti-GAPDH antibody, rabbit polyclonal anti-TLR3 antibody, rabbit polyclonal anti-NFκB antibody and mouse anti-MDV monoclonal antibody BD8 were used as primary antibodies, while either HRP-conjugated goat anti-mouse IgG or HRP-conjugated goat anti-rabbit IgG (Sigma-Aldrich, USA) served as the secondary antibody. Finally, the membranes were washed and visualized using chemiluminescence (Protein Simple, Fluorchem E FE0605).

### Statistical analysis

The data were analysed using the Student’s *t* test and GraphPad PRISM 6 software. P values <0.05 was considered significant.

## Abbreviations

IRF3: interferon regulatory factor3
IKKα: IκB kinase α
TANK: TRAF family member-associated NFκB activator
